# Engagement, Acceptability, and Effectiveness of the Self-Care and Coach-Supported Versions of the Vira Digital Behavior Change Platform Among Young Adults at Risk for Depression and Obesity: Pilot Randomized Controlled Trial

**DOI:** 10.2196/51366

**Published:** 2024-09-19

**Authors:** Lauren S Weiner, Ryann N Crowley, Lisa B Sheeber, Frank H Koegler, Jon F Davis, Megan Wells, Carter J Funkhouser, Randy P Auerbach, Nicholas B Allen

**Affiliations:** 1 Ksana Health Eugene, OR United States; 2 Center for Digital Mental Health University of Oregon Eugene, OR United States; 3 Oregon Research Institute Eugene, OR United States; 4 Integrated Physiology Research Global Drug Discovery Novo Nordisk Research Center Seattle Seattle, WA United States; 5 Department of Psychiatry Columbia University New York, NY United States; 6 Division of Child and Adolescent Psychiatry New York State Psychiatric Institute New York, NY United States; 7 Sackler Institute for Developmental Psychobiology Columbia University New York, NY United States

**Keywords:** depression, behavioral activation, digital health, mental health, behavior change, mobile sensing, anxiety, health coaching, mobile phone

## Abstract

**Background:**

Adolescence and early adulthood are pivotal stages for the onset of mental health disorders and the development of health behaviors. Digital behavioral activation interventions, with or without coaching support, hold promise for addressing risk factors for both mental and physical health problems by offering scalable approaches to expand access to evidence-based mental health support.

**Objective:**

This 2-arm pilot randomized controlled trial evaluated 2 versions of a digital behavioral health product, Vira (Ksana Health Inc), for their feasibility, acceptability, and preliminary effectiveness in improving mental health in young adults with depressive symptoms and obesity risk factors.

**Methods:**

A total of 73 participants recruited throughout the United States were randomly assigned to use Vira either as a self-guided product (Vira Self-Care) or with support from a health coach (Vira+Coaching) for 12 weeks. The Vira smartphone app used passive sensing of behavioral data related to mental health and obesity risk factors (ie, activity, sleep, mobility, and language patterns) and offered users personalized insights into patterns of behavior associated with their daily mood. Participants completed self-reported outcome measures at baseline and follow-up (12 weeks). All study procedures were completed via digital communications.

**Results:**

Both versions of Vira showed strong user engagement, acceptability, and evidence of effectiveness in improving mental health and stress. However, users receiving coaching exhibited more sustained engagement with the platform and reported greater reductions in depression (Cohen *d*=0.45, 95% CI 0.10-0.82) and anxiety (Cohen *d*=0.50, 95% CI 0.13-0.86) compared to self-care users. Both interventions also resulted in reduced stress (Vira+Coaching: Cohen *d*=–1.05, 95% CI –1.57 to –-0.50; Vira Self-Care: Cohen *d*=–0.78, 95% CI –1.33 to –0.23) and were perceived as useful and easy to use. Coached users also reported reductions in sleep-related impairment (Cohen *d*=–0.51, 95% CI –1.00 to –0.01). Moreover, participants increased their motivation for and confidence in making behavioral changes, with greater improvements in confidence among coached users.

**Conclusions:**

An app-based intervention using passive mobile sensing to track behavior and deliver personalized insights into behavior-mood associations demonstrated feasibility, acceptability, and preliminary effectiveness for reducing depressive symptoms and other mental health problems in young adults. Future directions include (1) optimizing the interventions, (2) conducting a fully powered trial that includes an active control condition, and (3) testing mediators and moderators of outcome effects.

**Trial Registration:**

ClinicalTrials.gov NCT05638516; https://clinicaltrials.gov/study/NCT05638516

## Introduction

### Background

Adolescence and early adulthood are peak periods for the emergence of major depressive disorder [1-3] and other mental health problems [4,5]. In addition, health behaviors, both positive (eg, exercise and healthy diet) and negative (eg, substance use and unsafe sex practices), often become established during adolescence and frequently persist into adulthood [6].

Mental health disorders in adolescents and young adults often co-occur with risk factors for chronic physical diseases [7] and are strongly linked to poor physical health across the life span [8-10]. Although most research on the co-occurrence of mental and physical health problems has been conducted with adults [7], some studies have suggested that the initial comorbidity between mental and physical health begins to emerge during adolescence [7,11]. Depressive disorders in adolescence have been associated with later-onset physical diseases, including arthritis, allergies, heart disease, diabetes, digestive system diseases, and epilepsy [7]. Individuals with mental health diagnoses lose 10 years from their lives, on average, due to all-cause mortality [12], and the predictive power of mental health disorders on these outcomes is similar to that of other well-known biomedical risk factors, such as smoking and obesity [10].

Moreover, mental health problems in young people often precede and predict the onset of risk factors for chronic physical health conditions [13]. This is especially true for obesity, a risk factor for a range of chronic diseases [14]. The National Longitudinal Study of Adolescent Health in the United States found that adolescents’ depressive symptoms were positively associated with risk for obesity 1 year later, even among those not obese at baseline [15]. Similarly, the Coronary Artery Risk Development in Youth Adults study found that young adults aged 18 to 30 years with higher levels of self-reported depressive symptoms experienced greater increases in their BMI and waist-to-hip ratio [16]. Accordingly, adolescents and young adults experiencing depression are an ideal target group for a risk reduction approach.

Behavioral activation (BA) is an evidence-based treatment for depression [17,18]. It has also been shown to improve a range of other depression-related phenomena, such as anxiety [19], sleep [20], emotional and social support [21,22], obesity [23], and general well-being, among those without clinical diagnoses [24]. In BA, people learn techniques to monitor their mood and daily activities, observe the connection between them, and then develop a plan to increase the number of pleasant activities and positive interactions with their environment. Importantly, the types of positive behavior change targeted by BA (eg, increasing physical activity and increasing social connection) can not only improve mood but are also strong inverse correlates of risk for chronic disease [25-27]. Thus, BA is an intervention that may reduce risk for chronic disease in two ways: (1) by reducing mental health–related risk factors and (2) by instigating positive behavior changes that can directly impact risk factors for obesity and related disorders.

BA can also be easily adapted to a brief intervention format [28] and can be delivered digitally [29], increasing its potential for scalability. Scalability is important because the traditional model of delivery (eg, face-to-face treatment) cannot meet the rising mental health needs of the population [30]. A recent meta-analysis revealed that digital BA interventions, used as self-guided tools or with support from a therapist or lay coach, can improve depressive symptoms, anxiety, and other aspects of quality of life [31]. Thus, using digital methods to deliver interventions such as BA is a promising approach to expanding access to evidence-based mental health support.

One of the most pressing issues for digital interventions is establishing evidence of effectiveness, as well as user adherence, typically a prerequisite to effectiveness [32]. Self-guided or stand-alone digital mental health apps, which are fully technological in their delivery, show great promise in terms of their scalability [33]. However, some meta-analyses have raised questions about whether self-guided apps are effective in improving mental health [34,35]. Other reviews have concluded that interventions incorporating human support (eg, coaching, manual adherence prompts, or integration with professional face-to-face care) yield greater effectiveness [36,37] and adherence [38,39] than self-guided apps. If smartphone apps need human support to be effective, this resource limitation must be factored into the implementation of digital interventions. By contrast, if a self-guided app can show high adherence and effectiveness without human support, then the scalability of the approach is significantly enhanced.

One form of human support particularly relevant to community-based interventions is coaching that is designed to bolster the use of and engagement with the app [32]. With appropriate training, this type of coaching can be delivered successfully by nonspecialists, scheduled or on demand, and across various modalities (eg, phone, text, and social media) [40]. Previous studies have found that coaching can contribute to the effectiveness of digital mental health interventions across many use cases [41-43] and that users are interested in coaching support when using an app [44,45].

### Goals of This Study

The purpose of this 2-arm pilot randomized controlled trial was to assess the feasibility, acceptability, and preliminary effectiveness of 2 versions of a digital BA product (Vira, Ksana Health Inc) in young adults with depressive symptoms and obesity risk factors. Users were randomly assigned to use Vira as a self-guided product (Vira Self-Care) or Vira with support from a trained health coach (Vira+Coaching) for 12 weeks. The Vira smartphone app includes functions that leverage the passive sensing of behavioral data related to mental health (ie, data on activity, sleep, mobility, and language patterns) and provides feedback in the form of personalized insights about patterns of behavior associated with their daily mood. These functions target positive behavior changes that can both reduce mental health symptoms (including depression and anhedonia) and directly impact risk factors for obesity and related disorders (eg, increased physical activity, improved sleep quality, increased mobility, and improved mental health [46-49]). The primary aims were to examine the following: (1) feasibility of and engagement with the platform, defined as objectively measured daily active use and retention, as well as self-reported use of Vira features; (2) acceptability of the platform, operationalized as ratings on items adapted from the Technology Acceptance Model (TAM) [50] and user feedback; and (3) preliminary evidence of clinical effectiveness, assessed through change in depressive symptoms from baseline to 3 months within and between study groups. Secondary aims were to evaluate the effectiveness of the 2 interventions on additional, self-reported aspects of mood and health (ie, anxiety, perceived stress, sleep-related impairment, sleep disturbance, and emotional support) by exploring preintervention to postintervention changes in these outcomes within and between study groups.

## Methods

### Study Design and Overview

This fully internet–based, 2-arm, pilot randomized controlled trial compared the feasibility, acceptability, and preliminary effectiveness of 2 versions of the Vira behavior change platform: a self-guided version (Vira Self-Care) versus a version with support from a health coach (Vira+Coaching). Participants were young adults with elevated depressive symptoms and obesity risk factors. The intervention period was 12 weeks long and was preceded and followed by web-based baseline and follow-up assessments, respectively. Randomization occurred after baseline assessment. Data were collected from November 2022 through May 2023. The trial was prospectively registered on ClinicalTrials.gov (NCT05638516).

### Ethical Considerations

All procedures were reviewed and approved by the Oregon Research Institute Institutional Review Board (Optimizing Vira). All coaches and study staff were trained in the ethical treatment of human participants in research. All participants received detailed information about the study and provided informed consent. Participants were compensated for assessment completion (US $30 gift card at baseline; US $40 at follow-up), regardless of app installation or use. Data were deidentified for analysis.

### Eligibility

Participants were eligible if they (1) were aged 18 to 25 years, (2) resided in the United States, (3) demonstrated English fluency and literacy, (3) had access to an Android (Google LLC) or iOS (Apple, Inc) smartphone, (4) reported elevated depressive symptoms (8-item Patient Health Questionnaire [PHQ-8] [51] score≥10), and (5) were overweight (BMI≥25 kg/m^2^) or reported a parental history of overweight or obesity. Participants were ineligible if they previously participated in the study.

### Procedures

#### Recruitment, Screening, and Informed Consent

Recruitment was conducted across the United States between November 2022 and January 2023, led by a market research firm, KJT Group Inc. The study was advertised to members of KJT’s existing databases and consumer research panels who have opted in to participate in the research. Research panels are advantageous in providing access to large population pools, enabling researchers to reach particular segments of the population and thus control sample composition [52]. In addition to eligibility requirements, recruitment was targeted at a nationally representative sample with regard to demographic characteristics.

Interested individuals completed a web-based screening questionnaire to assess eligibility. The screening questionnaire consisted of questions on demographic characteristics, self-reported height, weight, parental history of being overweight, and depressive symptoms (PHQ-8). Eligible individuals were presented with a web-based consent form and were contacted by a KJT staff member via phone to verify their eligibility and address questions related to the consent or study procedures. Contact information for eligible, verified, and consented individuals was sent to Ksana Health via a secure file sharing platform. No formal power calculations were conducted to determine the sample size for this pilot study.

#### Randomization and Onboarding

After completing the baseline assessment, participants were randomly allocated to either Vira Self-Care or Vira+Coaching. Participants were randomly allocated using a random number generation function (RANDBETWEEN) in Excel (Microsoft Corp). The random allocation sequence was generated by a biostatistician not involved in study enrollment or measurement. The biostatistician and project director assigned participants to conditions using the random allocation sequence. Assignment was completed in blocks of eligible participants. Upon assignment, each eligible participant was assigned a value from the random number generator, and allocation was determined based on gender and the random number. Per block, condition assignment was balanced by gender (equal; when possible, +1 or –1 was tracked across batches). Following randomization, participants in both conditions were emailed brief written directions on how to install and use the Vira app and subsequently completed a 5-minute phone call with the study coordinator to verify installation.

#### Intervention Conditions

Participants in both conditions had access to the Vira app for 12 weeks. During this period, they were prompted by the app to complete a single daily mood question with a 5-point rating scale: rate your enjoyment during the previous day, ranging from *not at all* to *super enjoyable!* They were also prompted to complete a daily, 3-item food questionnaire (results not presented here).

#### Vira Self-Care

**Figure 1:**
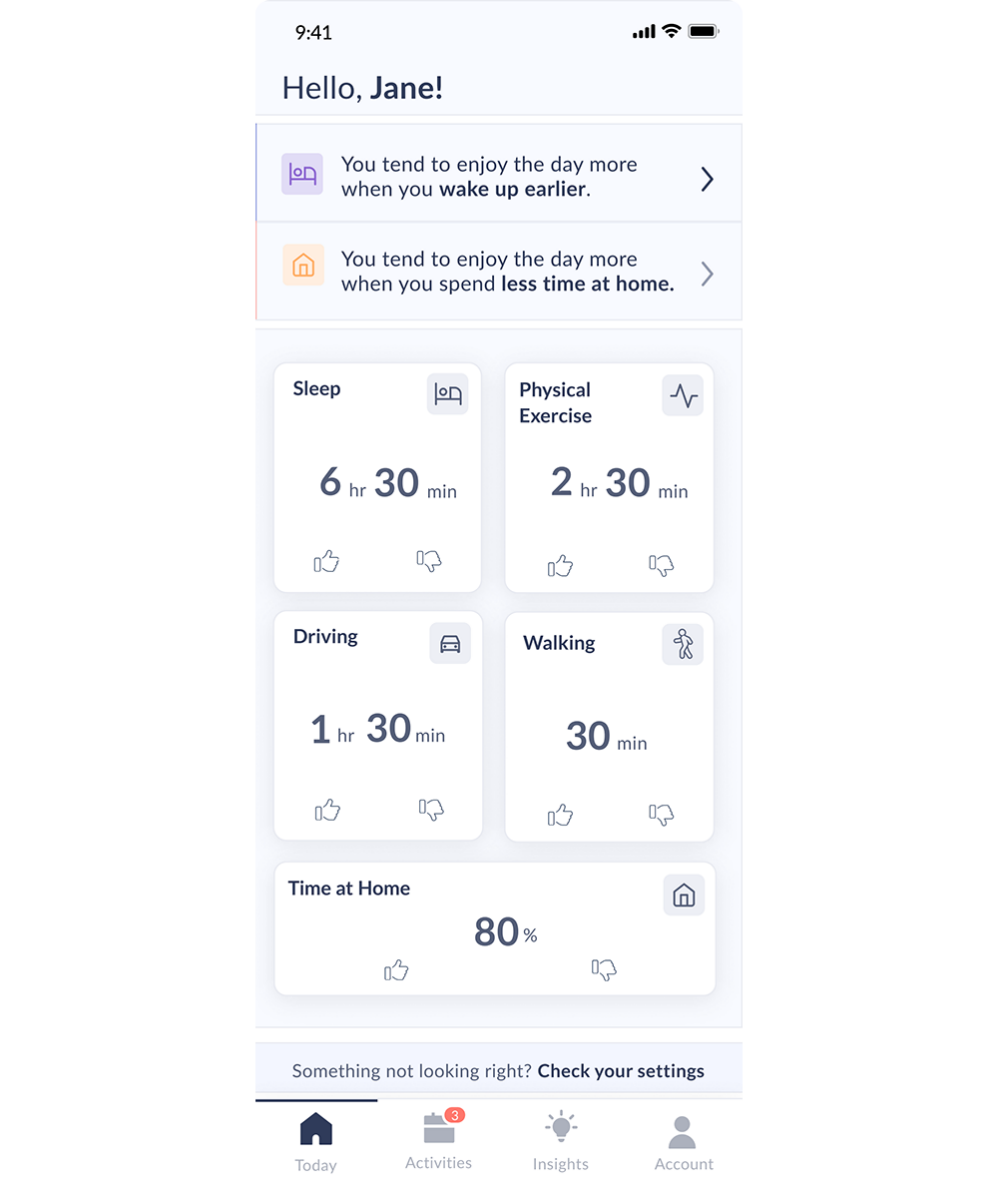
Weekly averages of the user’s passive sensing data and their most recent Vira insights are displayed on the Today screen of the Vira app.

**Figure 2:**
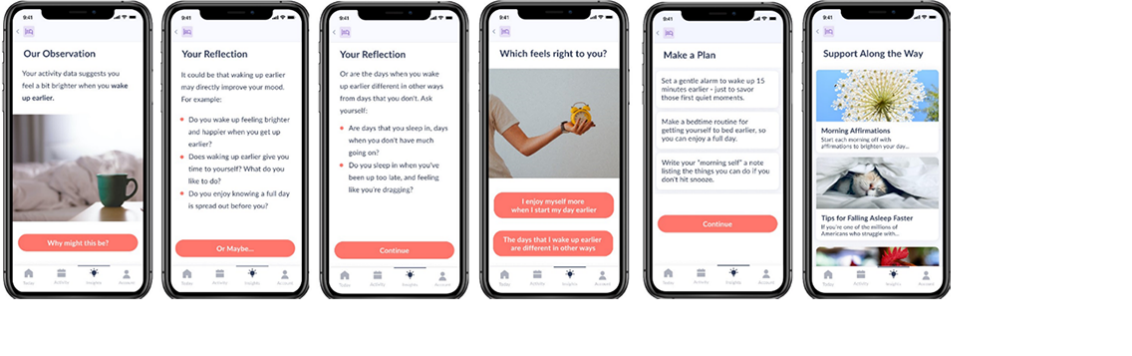
User experience flow of the Vira insights feature.

Participants in the Vira Self-Care condition used the Vira app as a self-guided intervention. The Vira app, installed on the participant’s smartphone, passively collected data indicative of risk-relevant behavioral patterns and psychological states from phone sensors (ie, measures of physical activity, sleep patterns, mobility, and language patterns reflecting mood states and cognition). Upon installation, participants were asked to grant the app permission to collect various data, which were then processed on their device using validated algorithms and displayed to the user on their *Today* (home) screen (Figure 1). The passive data streams collected by Vira have been shown to be related to changes in mental health [53-55]. The Vira app also prompted users to reflect on their sense of enjoyment over the previous day through one daily question. During an initial 10-day period, the app assessed the relationship between the user’s patterns of behavior and their day-to-day variations in mood and well-being using passive mobile sensing. After this period, the app continued to collect data and offered the user up to 2 personalized insights per week about behavioral factors (eg, sleep, movement, location, or communication patterns) that may relate to their mood. For example, Vira could suggest that “you seem to enjoy the days when you drive more” or “you tend to enjoy the day more when you wake up earlier.” The Vira app guided users to reflect on these insights and create a behavior change plan by exploring more detailed information about the insight (ie, insight details) and accessing in-app and external resources, such as links to articles about health behaviors and behavior change. The user experience for Vira insights is illustrated in Figure 2. In line with BA, pursuing activities that brought participants meaning or joy could break the cycle of thoughts and behaviors associated with poor mental health and improve their overall energy, mood, and quality of life [56]. Participants could also view their passive sensing data for the previous day (eg, bed and wake time, time away from home, time in transit, and movement time) and see a graphical display of their check-in ratings for the previous week (ie, weekly mood graph) within the Vira app.

#### Vira+Coaching

Participants in the Vira+Coaching condition used the same version of the Vira app as participants in the Vira Self-Care arm and additionally received text-based support and nudges within the Vira app related to their behavior change goals from a trained health coach. Table 1 lists differences between the coaching and self-guided versions of the Vira app experience. Coaches were trained in motivational interviewing techniques and followed a manualized approach to coaching. A total of 5 health coaches with diverse educational and professional backgrounds and a minimum of a bachelor’s degree (in any discipline) participated in the study. Of the 5 coaches, 2 (40%) had previous experience delivering behavioral interventions, but none were professional behavioral health providers (ie, none had degrees or training in a health profession, such as clinical psychology, psychiatry, or social work).

**Table 1:** Features of the coaching and self-care Vira app experiences.

Feature	Self-care version	Coached version
Prompt to complete daily mood check-in	✓	✓
Prompt to complete daily food questionnaire	✓	✓
In-app display of weekly data from passive mobile sensing	✓	✓
Personalized insights and in-app resources	✓	✓
Personalized nudges related to behavior change goals		✓
Interpersonal support from a human coach		✓

The primary role of the health coach was to support the participant in using Vira effectively and encourage engagement with the app. Through text conversations, coaches supported participants with usability or technical issues and encouraged consistent use of the app. They also facilitated engagement with BA-informed features of the Vira app. For instance, coaches supported participants in setting goals and creating action plans based on Vira’s personalized insights about the patterns of behaviors that may be impacting a user’s mood. Coaches encouraged participants to engage in activities that they found pleasurable or rewarding, such as physical, social, or self-care activities, and provided supportive accountability toward meeting their goals.

Coaches accessed a practitioner platform (Vira Pro) to monitor objective behavioral and self-report mood data weekly between sessions, schedule reminders (nudges), and provide encouragement and accountability to participants. In addition, coaches texted with participants through Signal (Signal Technology Foundation), a secure texting platform outside of the Vira app. Coaches were trained to follow a coaching manual and set a schedule for their coaching interactions; specifically, coaches were instructed to schedule weekly, synchronized text chats and to reach out to each participant at least once between weekly coaching sessions. Within these constraints, coaches were instructed to personalize the intervention for each participant based on participant preferences (eg, preference for more vs fewer reminders or nudges to practice techniques).

Participants scheduled a synchronized text chat with their coach approximately once per week. The initial coaching text session lasted approximately 45 to 60 minutes, with additional variability based on the participant’s response times and engagement in the conversation. The initial session focused on introductions, rapport building, participant’s motivations for joining, expectations for the Vira app and the coach’s role, and ways to optimize program participation. Subsequent weekly text chats lasted approximately 10 to 30 minutes and focused on helping participants stay on track with using Vira by identifying facilitators of and barriers to ongoing use and providing performance feedback to support progress toward behavior change goals. At the end of each session, coaches scheduled nudges through Vira Pro. Nudges arrived in the participant’s smartphone at scheduled times between weekly sessions to support their behavior change plan.

Coaches also monitored each participant’s progress in Vira Pro at least once per week between scheduled weekly text chats. Midweek monitoring, which could include additional nudges or texts, focused on providing accountability toward behavior change goals and encouraging consistent use of Vira. In the last week of the intervention, participants completed a final text chat. Final session topics included encouraging reflection on and the reinforcement of progress to date; strategies for maintaining behavior changes after coaching and access to Vira were no longer available; and providing logistical information about completing the study, including uninstalling the app and completing the follow-up assessment.

#### Assessments

Consented participants were invited to complete a battery of web-based questionnaires at baseline and again at follow-up after the 12-week intervention period (ie, 12 weeks after installing the Vira app). Assessments required 10 to 20 minutes to complete at baseline and 20 to 30 minutes to complete at follow-up. Participants received up to 4 reminders to complete the questionnaires at each time point. Participants who did not complete the follow-up assessment within 4 weeks of the end of the intervention period were considered lost to follow-up.

### Measures

#### Participant Characteristics

At baseline, participants self-reported their age, gender, race, ethnicity, education, and employment status. They also provided information about obesity risk factors, including self-reported height and weight and parental history of overweight.

#### App Feasibility and Engagement

Feasibility of and engagement with the Vira platform were measured through metrics derived from objective and self-reported use data. Objective use metrics included (1) the daily active use and (2) retention. Daily active use was operationalized as the percentage of users who completed the daily mood check-in and the daily food questionnaire per day. Retention was defined as the furthest day from installation on which active use was recorded for each participant (values range from 0 to 90) and is displayed as the percentage of the sample retained on a specific intervention day. Self-reported use metrics included the frequency of viewing one’s activity data, mood graph, insight details, and insight resources in the Vira app.

#### Acceptability

##### The TAM

Acceptability of the Vira platform was measured with 11 items based on the TAM [50]. The model centers on 2 specific beliefs shown to influence the acceptance of technology: perceived ease of use and perceived usefulness. Perceived ease of use was measured with a 10-item scale on which participants rated their experience using Vira on a 7-point Likert scale from 1 (strongly disagree) to 7 (strongly agree). Perceived usefulness was assessed with a single item about the usefulness of the app, using the same Likert scale as perceived ease of use items. Higher ratings indicated higher acceptability.

##### Follow-Up Feedback Questionnaire

At follow-up, participants were asked to rate the extent to which participating in the Vira program impacted them in three different ways: (1) changed them or their behavior, (2) increased their motivation to improve their mental health and well-being, and (3) increased their confidence in improving their mental health and well-being. Items were assessed on a 6-point Likert scale from 1 (strongly disagree) to 6 (strongly agree).

#### Effectiveness

##### The PHQ-8

The PHQ-8 is an 8-item self-report measure assessing the severity of depressive symptoms [51,57]. The PHQ-8 has high internal consistency (Cronbach α=0.89) and construct validity, with a score ≥10 used to define current depression [51].

##### Generalized Anxiety Disorder-7

The Generalized Anxiety Disorder-7 (GAD-7) is a 7-item self-report measure of the severity of anxiety symptoms, including excessive worry, restlessness, and difficulty concentrating. The GAD-7 has shown high internal consistency and convergent validity with other anxiety measures [58].

##### Perceived Stress Scale-10

The Perceived Stress Scale-10 (PSS-10) is a 10-item questionnaire that measures the degree to which various situations are perceived as stressful, capturing both subjective feelings and the perceived ability to cope with stress [59]. The PSS-10 has shown good reliability and validity [60].

##### Patient-Reported Outcomes Measurement Information System Measures

Sleep disturbance, sleep-related impairment, and emotional support were assessed via Patient-Reported Outcomes Measurement Information System (PROMIS) short form self-report measures for each construct (Sleep Disturbance, Sleep-Related Impairment, and Emotional Support, respectively) [61-63]. The PROMIS sleep disturbance and sleep-related impairment measures have demonstrated excellent measurement properties across various patient groups and interventions [64]. Each PROMIS measure yields a standardized *t* score with a mean of 50 and an SD of 10. Higher scores indicate higher levels of the construct.

#### Analytic Plan

##### Participant Characteristics

Descriptive statistics and frequencies were used to examine participant characteristics within and across study arms. Chi-square and 2-sample 2-tailed *t* tests were used to examine between-arm differences in baseline characteristics. Overall study attrition and differential study attrition (ie, after assessment completion) between study arms were also calculated.

##### Feasibility and Engagement

Analyses of feasibility and engagement were restricted to users who downloaded the Vira app and connected to a coach for coached users or a study coordinator for self-care users. Metrics were evaluated per study arm and are reported using frequencies and descriptive statistics. Linear regression and Fisher exact tests were used to evaluate the effects of study arm on objectively measured days of active use and self-reported use of Vira features, respectively. Of the 35 self-care users, 4 (11%) reported never seeing data on the Vira app home screen and were excluded from analyses of that variable. Overall, 4 (10%) of the 38 coached users and 2 (6%) of the 35 self-care users reported never receiving insights in the Vira app and were excluded from analyses of insight features. Data were analyzed using the R software (version 4.1.3; R Foundation for Statistical Computing).

##### App Acceptability

App acceptability was assessed through items adapted from the TAM and the follow-up feedback questionnaire. Analyses were restricted to users who downloaded the Vira app and completed the follow-up assessment. The effects of study arm on acceptability ratings from the adapted TAM were evaluated using separate linear regression models for each outcome (perceived ease of use and perceived usefulness). For the analysis of the follow-up feedback items (ie, the extent to which Vira changed participants or their behavior, increased their motivation, and improved their confidence), response options were dichotomized into 2 categories representing the top half (strongly agree, agree, and slightly agree) and bottom half (slightly disagree, disagree, and strongly disagree) of the Likert scale. Separate Fisher exact tests were used to evaluate the effect of study arm on each feedback item. Data were analyzed using the R software.

##### Effectiveness

Descriptive statistics and frequencies were calculated at each time point (baseline and 12 weeks) for each outcome: PHQ-8, GAD-7, PSS-10, PROMIS sleep disturbance, PROMIS sleep-related impairment, and PROMIS emotional support. An intention-to-treat approach was used to avoid the overestimation of intervention effects on patient-reported outcomes. Multiple imputation was used to account for missing outcome data at the 12-week time point. Little's Missing Completely at Random test [65] was used to assess whether data were missing at random or missing completely at random.

Separate linear mixed-effects regression models were used to examine between-group differences over time for each outcome. Each model included fixed-effect terms for group (coaching vs self-care), time point (baseline and 12 weeks), and the time-by-group interaction. To account for the correlation between repeated measures of the same individuals over time, each model included a participant-level random intercept. Differences in outcome scores within each group over time were explored using linear mixed-effects models. Each model included a fixed-effect term for time point (baseline and 12 weeks) and a participant-level random intercept. Data were analyzed using the *lme4* package (version 1.1.233) [66] in the R software.

## Results

### Participant Characteristics and Study Flow

**Figure 3:**
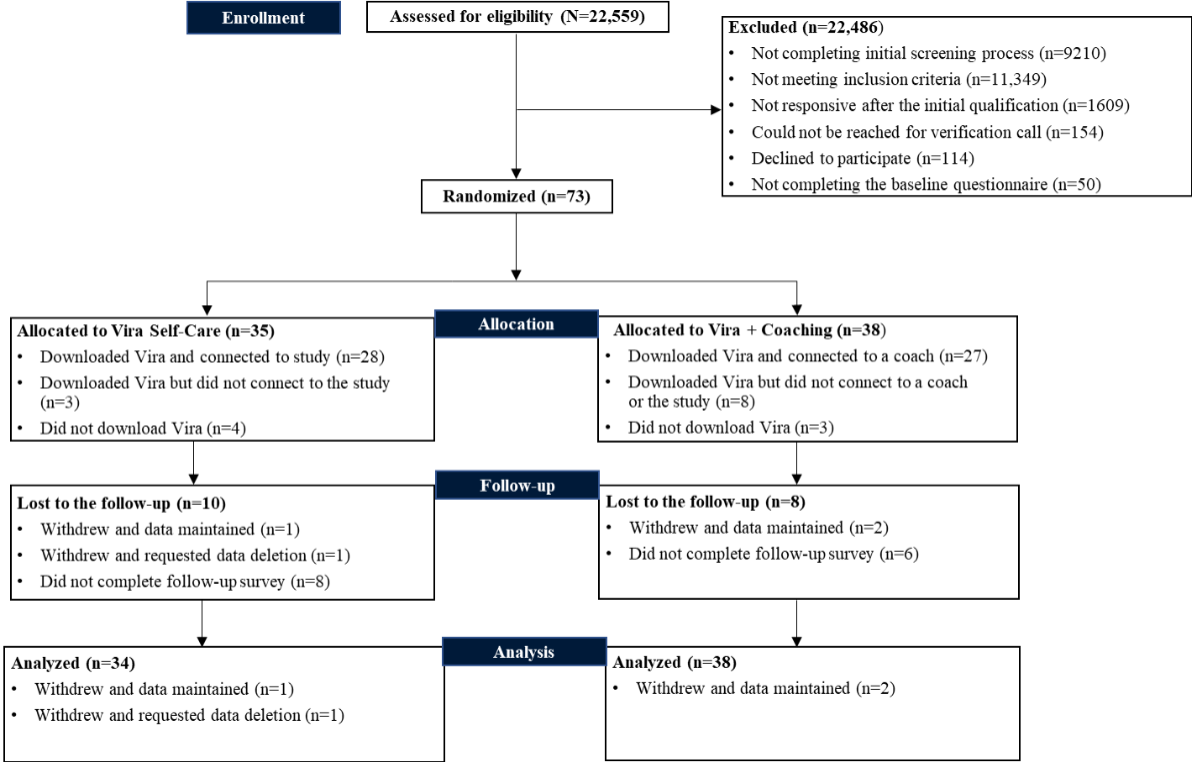
CONSORT (Consolidated Standards of Reporting Trials) diagram.

A total of 73 individuals completed the baseline assessment and were randomly assigned to either the Vira+Coaching (n=38, 52%) or Vira Self-Care (n=35, 48%) arm. Figure 3 presents the CONSORT (Consolidated Standards of Reporting Trials) diagram. Overall, 35 (92%) of the 38 coached participants and 31 (91%) of the 35 self-care participants downloaded the Vira app. Of the total 73 participants, 18 (25%; coached: 8/18, 44%; self-care: 10/35, 29%) were lost to follow-up, resulting in an overall attrition rate of 25% (18/73; coached=8/38, 21%; self-care=10/35, 29%) and a differential attrition rate of 7.5% (no significant difference between study arms; *P*=.59). Demographic characteristics of study participants at baseline, stratified by arm, are provided in Table 2. There was a significant difference in average age at baseline between the coached and self-care groups (*P*=.006). No other significant between-group differences in demographic characteristics emerged (*P*>.05).

**Table 2:** Baseline characteristics by study arm.

Demographic characteristics		Vira+Coaching (n=38)	Vira Self-Care (n=35)	Overall (N=73)
Age (y), mean (SD)		21.2 (2.2)	22.6 (1.0)	21.9 (2.2)
BMI (kg/m^2^), mean (SD)		30.3 (8.7)	28.4 (7.2)	29.4 (8.0)
**Overweight status, n (%)**				
	Self-overweight	25 (66)	20 (57)	45 (62)
	Parental history of overweight	32 (84)	29 (83)	61 (84)
	Self and parental history of overweight	19 (50)	14 (40)	22 (45)
**Gender, n (%)**				
	Cisgender female	25 (66)	23 (66)	48 (66)
	Cisgender male	9 (24)	9 (26)	18 (25)
	Transgender female	1 (3)	1 (3)	2 (3)
	Transgender male	0 (0)	1 (3)	1 (1)
	Nonconforming	3 (8)	1 (3)	4 (5)
**Race, n (%)^a^**				
	African American	10 (26)	8 (23)	18 (25)
	Asian American	3 (8)	4 (11)	7 (10)
	White	28 (74)	21 (60)	49 (67)
	Other	1 (3)	1 (3)	2 (3)
Hispanic, n (%)		5 (13)	6 (17)	11 (15)
**Operating system, n (%)**				
	Android	18 (47)	15 (43)	33 (45)
	iOS	20 (53)	20 (57)	40 (55)
Depressive symptoms (PHQ-8^b^), mean (SD)		14.8 (5.1)	14.0 (4.3)	14.4 (4.7)
Anxiety symptoms (GAD-7^c^), mean (SD)		13.9 (5.6)	13.7 (4.7)	13.8 (5.2)

^a^The totals across the categories are higher than the group sizes because participants could self identify with more than one racial identity.

^b^PHQ-8: 8-item Patient Health Questionnaire.

^c^GAD-7: Generalized Anxiety Disorder-7.

### App Feasibility and Engagement

**Figure 4:**
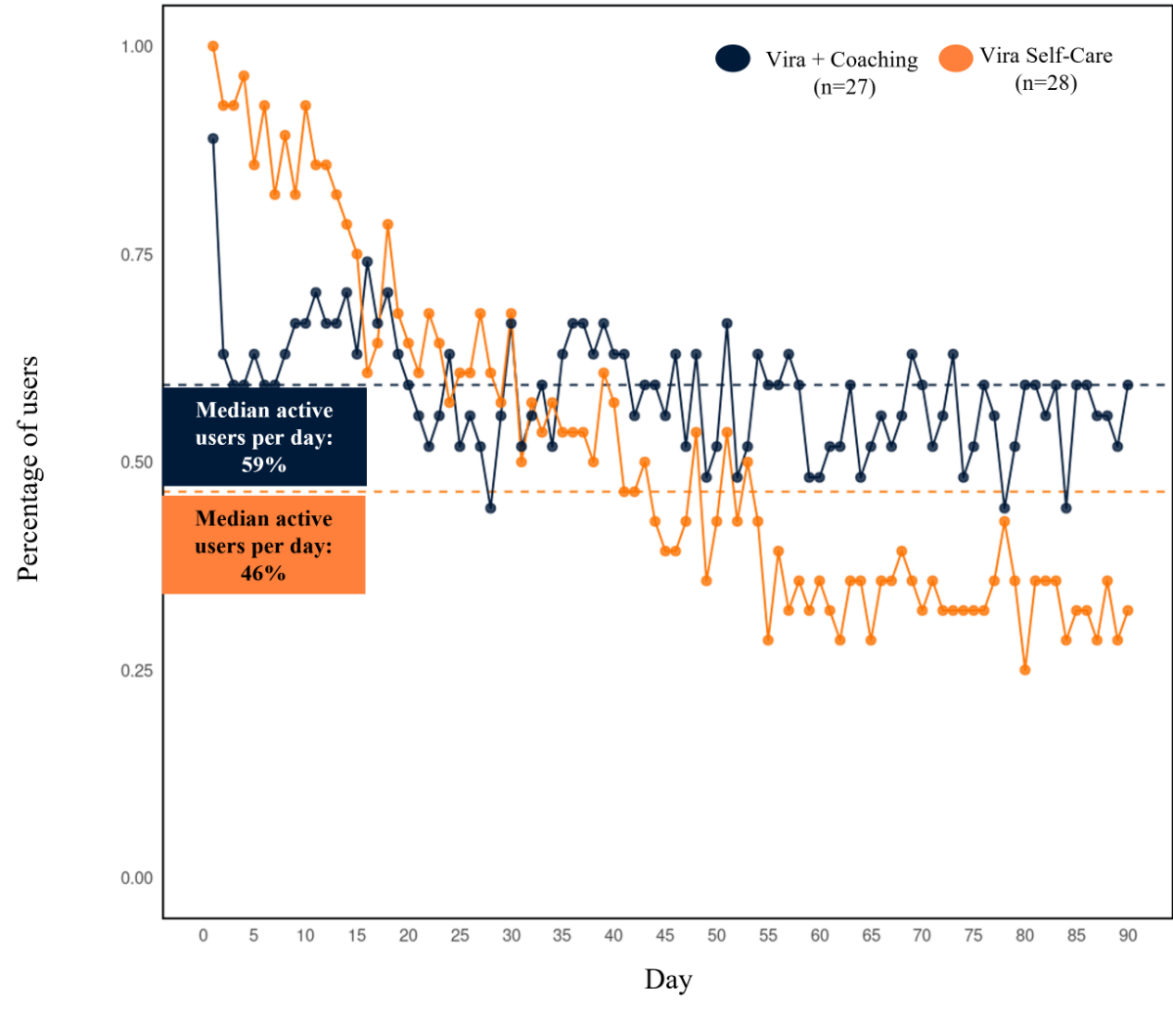
Daily active use over the 90-day intervention period by study arm. Users who downloaded Vira and connected it to the trial (55/73, 75%) are included. Actively engaged=completed daily mood check-in or food questionnaire.

**Figure 5:**
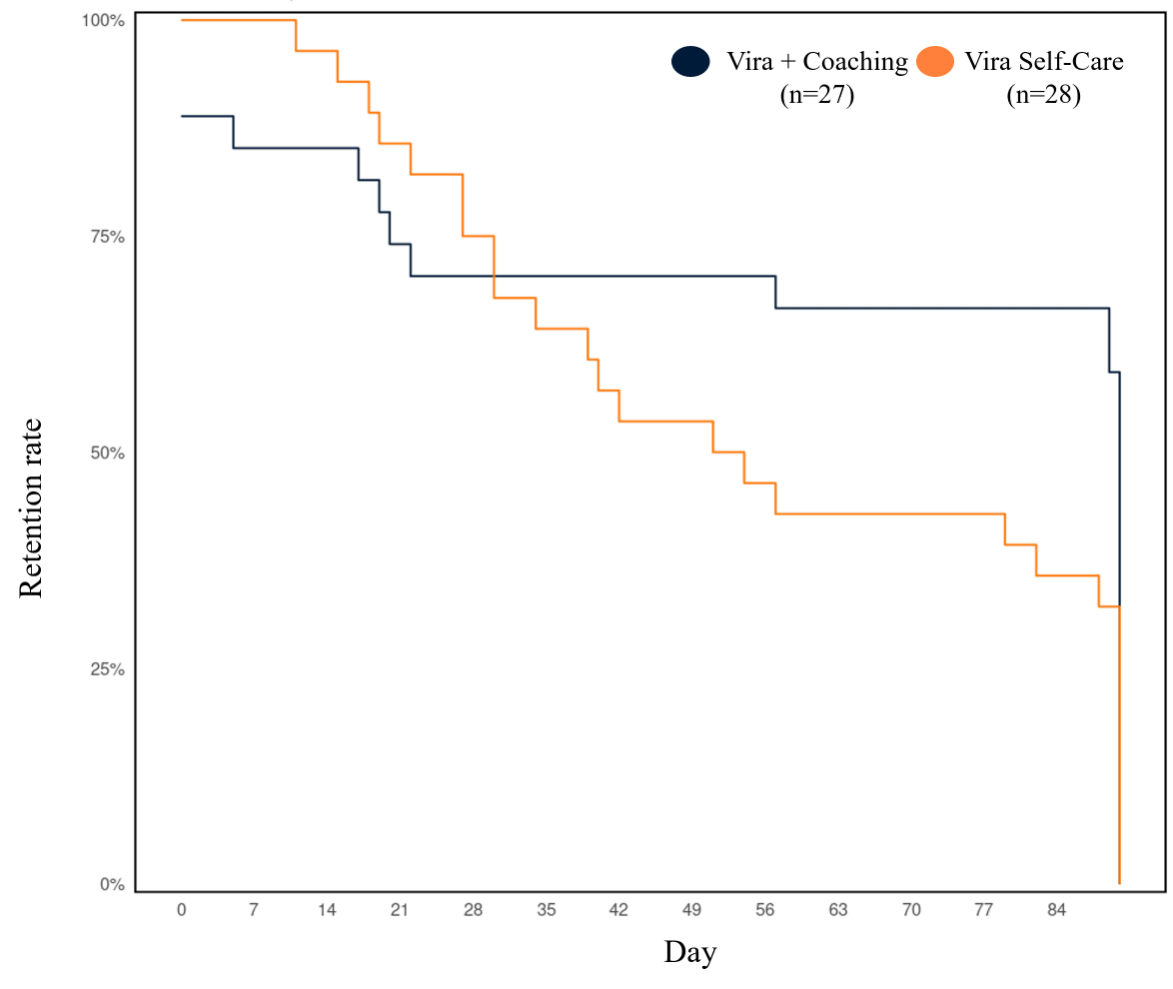
User retention by the length of time in study. Users who downloaded Vira and connected it to the trial (55/73, 75%) are included. Retention=last active day of use across the 90-day intervention period. Values range from 0 to 90, with app installation occurring on day 0.

The percentage of active users per day over the 90-day intervention period is displayed in Figure 4. Among participants who downloaded the Vira app and connected it to the study, those allocated to Vira+Coaching had a median of 63 (IQR 15-79.5) active days (63/90, 70% of intervention days), and Vira Self-Care users had a median of 45.5 (IQR 21.8-75) active days (45.5/90, 51% of intervention days); the number of active days did not significantly differ between groups (β=–12.67, SE=8.05; *t_50_*=–1.58, *P=*.12). Overall, 30% (8/27) of users engaged with the app on <30% (26/90) of intervention days in the Vira+Coaching group, whereas 32% (9/28) of users engaged with the app on <30% (26/90) of intervention days in the Vira Self-Care group. Retention rates among both coached and self-care users declined across the first 3 weeks of the intervention period. The retention of coached participants remained relatively stable for the remainder of the 90-day intervention period, whereas the retention of self-care participants continued to decline over time (Figure 5).

**Figure 6:**
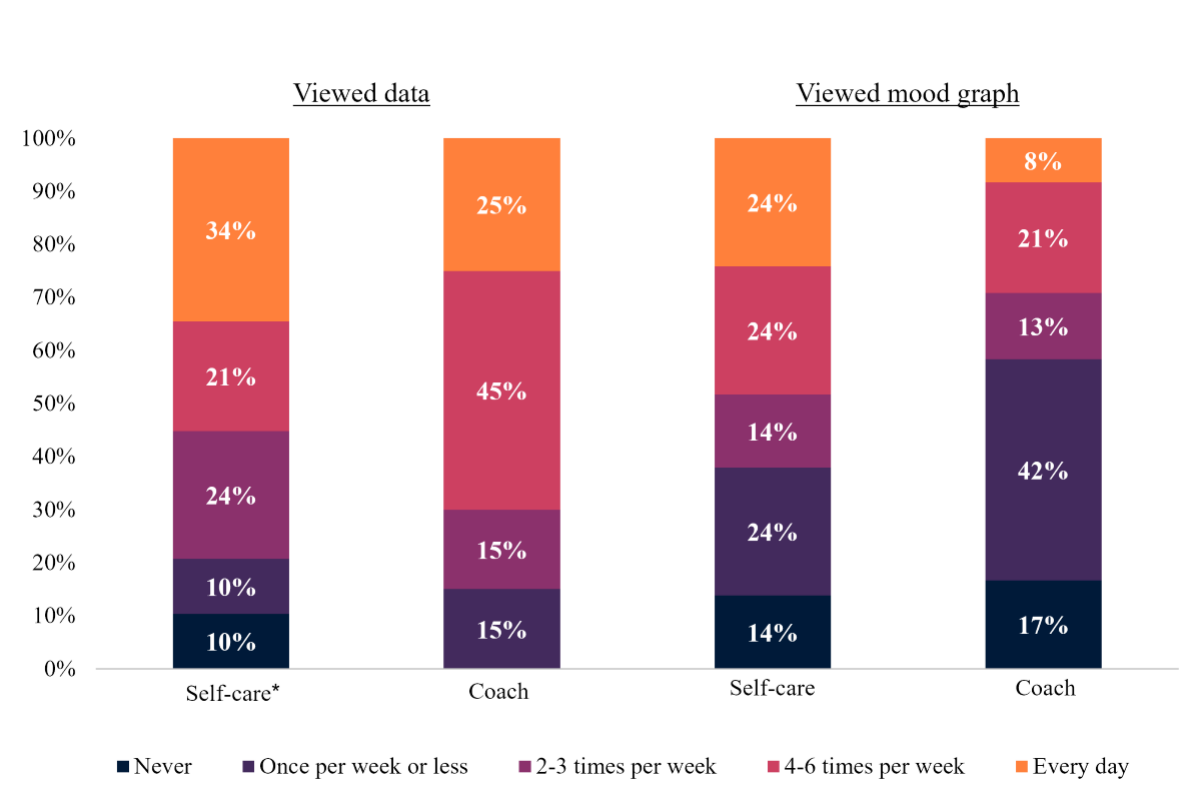
Self-reported engagement with Vira features. Users who downloaded Vira, connected it to the trial, and completed the postintervention assessment (coached: 29/38, 76%; self-care: 24/35, 69%) are included. A total of 4 self-care users reported never seeing data on the Today screen and were excluded from the analysis of this variable.

Participants in both the coached and self-care groups reported high engagement with Vira features (Figure 6). There were no significant between-group differences in the frequency of viewing one’s activity data (*P*=.30) or the weekly mood graph (*P*=.52). Users also reported high engagement with insights features. All coached users and 96% (21/22) of self-care users who reported receiving a Vira insight endorsed viewing an insight’s details at least once (*P*=.46). A greater proportion of coached than self-care users reported viewing psychoeducational resources upon receiving an insight at least once, although this difference was not statistically significant (Vira+Coaching: 22/25, 88%; Vira Self-Care: 14/22, 64%; *P*=.08; odds ratio [OR] 4.06, 95% CI 0.80-27.82).

### App Acceptability

Coached and self-care participants reported similar ratings of acceptability, such that there were no significant group effects on perceived ease of use (β=.13, SE=1.29; *t_51_*=0.10, *P*=.92; Cohen *d*=–0.03, 95% CI –0.57 to 0.51), or perceived usefulness (β=–.19, SE=0.39; *t_51_*=–0.49, *P*=.63; Cohen *d*=0.13, 95% CI –0.41 to 0.68). Ratings for individual perceived ease of use and perceived usefulness items are displayed in Table 3. Moreover, a similar proportion of users across groups felt that participating in the Vira program changed themselves or their behavior (Vira+Coaching: 20/29, 69%; Vira Self-Care: 17/24, 71%; *P*>.99; OR 0.92, 95% CI 0.23-3.47) and increased their motivation to improve their mental health (Vira+Coaching: 23/29, 79%; Vira Self-Care: 17/24, 71%; *P*=.53; OR 1.56, 95% CI 0.37-6.78). A greater proportion of coached users than self-care users reported increased confidence in their ability to improve their mental health (Vira+Coaching: 24/29, 83%; Vira Self-Care: 13/24, 54%; *P*=.04; OR 3.95, 95% CI 1.00-17.88).

**Table 3:** Mean Vira acceptability ratings by study group. Includes users who downloaded Vira and completed Technology Acceptance Model items at follow-up (53/73, 73%).

		Vira+Coaching, mean (SD)	Vira Self-Care, mean (SD)
**Perceived ease of use**			
	Easy to use	6.1 (1.1)	6.0 (1.4)
	Easy to become skillful	5.8 (1.4)	5.5 (1.6)
	Easy to understand	6.1 (1.1)	6.2 (0.9)
	Mentally effortful	2.7 (1.7)	3.2 (1.6)
	Easy to remember how to use	5.9 (1.1)	5.8 (1.5)
	Rigid and inflexible	2.9 (1.8)	2.8 (1.8)
	Confusing	2.3 (1.7)	2.2 (1.4)
	Frustrating	2.6 (2.0)	2.5 (1.8)
	Easy to learn	5.9 (1.3)	6.0 (1.2)
	Cumbersome	3.4 (1.8)	3.5 (1.5)
Perceived usefulness		5.5 (1.5)	5.3 (1.3)

### Effectiveness

Effectiveness data were imputed for 17 participants who were missing follow-up assessments. Little's test revealed that data were missing at random and not missing completely at random (*χ*^2^_11_=23.2; *P*=.02). Changes in patient-reported outcomes from baseline to 12 weeks within and between the coaching and self-care arms are presented in Table 4. Reductions in depressive symptoms were significantly greater in the coaching arm than in the self-care arm, with a large effect size for the coached group (Cohen *d*=–1.07, 95% CI –1.59 to –0.55) versus a small, nonsignificant effect size for the self-care group (Cohen *d*=–0.32, 95% CI –0.85 to 0.21; Table 4). Coached participants also reported significantly greater reductions in anxiety symptoms (GAD-7) than self-care participants, with a large effect size for the coached group (Cohen *d*=–1.02, 95% CI –1.53 to –0.50) versus a small, nonsignificant effect size for the self-care group (Cohen *d*=–0.12, 95% CI –0.65 to 0.41; Table 4). No further group × time differences emerged for perceived stress, sleep-related impairment, sleep disturbance, or emotional support.

**Table 4:** Change and differences in patient-reported outcomes at baseline and follow-up (12 weeks) by study arm (N=73).

Variable and group		Within-group change over time						Difference in change between groups				
		Baseline, mean (SD)	Follow-up, mean (SD)	Change	*P* value	Effect size (95% CI)	Estimate (SE)		*P* value		Effect size, (95% CI)	
**PHQ-8^a^**								4.01 (1.58)		.01		0.45 (0.10 to 0.82)
	Coaching	14.76 (0.88)	9.58 (0.98)	–5.19 (1.21)	<.001	–1.07 (–1.59 to –0.55)						
	Self-care	14.15 (0.74)	13.00 (0.85)	–1.15 (0.97)	.24	–0.32 (–0.85 to 0.21)						
**GAD-7^b^**								4.39 (1.61)		.007		0.50 (0.13 to 0.86)
	Coaching	13.92 (0.94)	9.04 (1.04)	–4.88 (1.19)	<.001	–1.02 (–1.54 to –0.50)						
	Self-care	13.56 (0.83)	13.08 (0.95)	–0.48 (1.04)	.65	–0.12 (–0.65 to 0.41)						
**PSS-10^c^**								2.68 (1.58)		.09		0.31 (–0.05 to 0.67)
	Coaching	26.11 (0.91)	20.86 (1.02)	–5.24 (1.24)	<.001	–1.05 (–1.57 to –0.53)						
	Self-care	27.09 (0.90)	24.53 (0.99)	–2.56 (0.88)	.005	–0.78 (–1.33 to –0.23)						
**PROMIS^d^ Sleep Disturbance 4a**								1.91 (1.28)		.14		0.27 (–0.09 to 0.63)
	Coaching	53.47 (0.55)	52.63 (0.62)	–0.85 (0.71)	.24	–0.30 (–0.78 to –0.20)						
	Self-care	53.14 (0.94)	54.22 (1.07)	1.07 (1.10)	.33	0.26 (–0.27 to 0.79)						
**PROMIS Sleep-Related Impairment 4a**								2.58 (2.87)		.37		0.16 (–0.19 to 0.58)
	Coaching	63.06 (1.66)	58.45 (1.87)	–4.61 (2.27)	.046	–0.51 (–1.00 to –0.01)						
	Self-care	64.46 (1.41)	62.58 (1.58)	–1.87 (1.56)	.23	–0.32 (–0.85 to 0.21)						
**PROMIS Emotional Support 4a**								2.00 (2.41)		.41		0.15 (–0.21 to 0.51)
	Coaching	46.40 (1.57)	49.02 (1.68)	2.62 (1.38)	.06	0.48 (–0.02 to 0.97)						
	Self-care	44.52 (1.55)	49.09 (1.78)	4.58 (2.01)	.03	0.61 (0.07 to 1.15)						

^a^PHQ-8: 8-item Patient Health Questionnaire.

^b^GAD-7: Generalized Anxiety Disorder-7.

^c^PSS-10: Perceived Stress Scale-10.

^d^PROMIS: Patient-Reported Outcomes Measurement Information System.

Within-group models indicated that participants in both the coaching and self-care arms reported significant reductions in perceived stress over time, with medium-to-large effect sizes for both groups (Vira+Coaching: Cohen *d*=–1.05, 95% CI –1.57 to –0.50; Vira Self-Care: Cohen *d*=–0.78, 95% CI –1.33 to –0.23). Furthermore, the coaching arm reported significant reductions in sleep-related impairment from baseline to 12 weeks with a medium effect size (Cohen *d*=–0.51, 95% CI –1.00 to –0.01). Within the self-care group, there was a significant increase in emotional support from baseline to 12 weeks (Cohen *d*=0.48, 95% CI –0.02 to 0.97). There were no significant within-group differences in change over time for sleep disturbance. Table 4 provides full results.

## Discussion

### Principal Findings

This is the first pilot randomized controlled trial to examine the feasibility, acceptability, and effectiveness of a BA-focused digital health product in young adults with elevated depressive symptoms and risk for obesity. Both versions of the app demonstrated high engagement and acceptability. Users reported that the Vira platform was useful and easy to use, which likely fostered their motivation and confidence to enact behavioral changes. Users receiving coaching alongside the Vira app exhibited more sustained engagement than those who used the app on their own. The intervention provided by the Vira platform was effective, with those receiving coaching reporting larger reductions in depression and anxiety than self-care users. Both intervention conditions resulted in reductions in stress, and coached users reported less sleep-related impairment.

### Engagement and Acceptability

Both coached and self-care users showed strong and sustained engagement with Vira. Coached users engaged on a greater number of intervention days than self-care users (median 63, IQR 15-79.5 vs 45.5, IQR 21.8-75), and there was a higher median proportion of active users per day in the coaching group than in the self-care group, although differences between conditions were not statistically significant. The engagement rates in this study exceeded those observed in many trials of app-based interventions, although use is likely to be lower outside of a trial context. For example, Baumel and Kane [67] found that the median daily use rate for real-world depression apps was 4.8%. A subsequent review of studies that proactively recruited users and included pre-to-post assessment comparisons (ie, a clinical trial context) revealed that the median digital mental health intervention use was 4.06 times higher than the real-world use of the same intervention [68]. The objectively measured engagement rates observed in both the self-care and coaching conditions in this study exceeded both these industry benchmarks. Participants in both conditions also self-reported frequent use of Vira product features, and there were similar ratings of perceived usefulness and ease of use in the coaching and self-care groups. Taken together, these findings provide compelling evidence of Vira’s feasibility and use.

The completion of the daily check-in item and food questionnaire was used as the objective metric of daily engagement because these were the only user actions that were expected to be performed daily and that could be objectively tracked in the app at the time of the study. While there is no universally accepted and comprehensive definition of engagement with mental health interventions, it has been suggested that engagement is a multifaceted construct that includes user interactions with an app’s user interface and user experience and engagement with the behavior change intervention components and active ingredients specifically designed to influence behavioral determinants, which in turn impact behavior change [69-71]. Therefore, we also analyzed user engagement with Vira features designed to support BA, such as viewing personalized behavioral insights and resources. However, these data were collected via self-report, and participants were not queried about all BA-related app interactions (eg, viewing a nudge or reminder related to their behavior change goal). Since the study was performed, additional automated tracking functionality has been added to the Vira platform. Future studies of Vira will benefit from a more robust analysis of objective engagement metrics representing user engagement with both user interface and user experience components and behavior change intervention components that support BA and behavior change. In particular, engagement metrics aligned with behavior change intervention components will allow further exploration of mechanisms of action, both for behavior change and improvements in clinical outcomes, and support continued optimization of the Vira platform and its implementation.

Notably, self-care users demonstrated higher engagement early in the intervention, but their engagement was less sustained than that of coached users. The early advantage for self-care users is likely attributable to the lower friction associated with onboarding to an app-only experience versus having to coordinate with a coach to get started. Efficient access to digital mental health apps without waiting time is particularly important for high-risk populations, given that depression is associated with both (1) high levels of negative affect that lead to people searching for things to try in periods of escalating distress and (2) difficulties with sustained motivation and energy [72,73]. This finding also speaks to the relative benefits of the 2 approaches. Self-care users appear to have found it easier to navigate their initial engagement, which they could manage independently, whereas coached users found it easier to sustain their engagement across the 12-week intervention period.

In terms of acceptability, participants across conditions reported that they found the intervention to be useful and acceptable, with few differences between the conditions. Users reported that the platform helped them change their behavior, increased their confidence in improving their mental health and motivation to improve their mental health, and helped them learn about their behavior. In the coaching condition, nearly all participants reported that working with a coach motivated them to change their behavior and improve their well-being.

### Effectiveness

The primary effectiveness outcome for this study was depressive symptoms as measured by the PHQ-8. Coached users experienced greater reductions in depressive and anxiety symptoms than self-care users after 12 weeks of use. Moreover, the magnitude of changes observed for these outcomes in the coached group is on par with established minimally clinically important differences [74]. The between-group effect sizes reported in this study for depressive symptoms (Cohen *d*=0.45) and anxiety symptoms (Cohen *d*=0.50) are consistent with meta-analyses of mobile apps for mental health issues and a meta-analysis of 28 studies of traditional (ie, nondigital) BA treatments, with some variability based on the comparator (eg, waitlist control, active control, or inactive control [19,75]).

With regard to other secondary outcomes, both coaching and self-care groups reported reductions in perceived stress and sleep-related impairment (ie, alertness, sleepiness, and tiredness during usual waking hours). By contrast, sleep disturbance (ie, sleep quality, depth, and perceived difficulties related to getting and staying asleep) did not significantly change across the intervention period. We also observed that both groups experienced an improvement in their experiences of emotional support, although this improvement was only statistically significant for self-care users. Overall, these effect sizes are useful for powering and guiding future trials to further understand how the Vira platform impacts mental health outcomes.

The findings that coached users showed more sustained engagement (albeit not a significantly greater number of active days) and greater improvements in depressive and anxiety symptoms than self-care users are consistent with literature suggesting that human support enhances the adherence to [38] and efficacy of [34] digital mental health interventions. In a similar trial comparing coached versus unguided (ie, self-care) treatment with a digital mental health app (with or without weekly app recommendations), Mohr and colleagues [42] found that coaching resulted in more app downloads but not a greater number of app sessions or increased use. Participants in both the coached and self-guided arms of that trial showed significant reductions in depressive (9-item Patient Health Questionnaire) and anxiety (GAD-7) symptoms over time, with significantly stronger effects for the coached group in anxiety (although not depressive) symptoms. Moreover, participants who received weekly app recommendations (similar to the nudges sent by coaches in the Vira+Coaching condition) showed greater improvement in depressive symptoms than those who were told to browse the available apps. This study is also not the first to evaluate a digital platform that uses mobile sensing data to personalize treatment components. Frank and colleagues [76] reported a trial with Cue, a digital intervention that uses mobile sensing data to provide participants with personalized microinterventions based on social rhythm therapy. Their trial found that the combination of Cue with treatment as usual was not more effective than digital monitoring alone with treatment as usual for treating depression, but a subgroup analysis did show a stronger effect of the Cue intervention in a subgroup with moderately severe to severe depression. Currey and Torous [77] reported on a study using the MindLAMP app, which used mobile sensing data to provide algorithm-recommended personalized app interventions. Participants were allocated to conditions where they received (1) an automated email signed by a human research assistant encouraging them to complete an additional activity based on their data, (2) a similar activity suggestion from a study bot (Marvin), or (3) no additional activity suggestions. Outcome evaluations, however, did not detect any differences between the 3 conditions in terms of the number of activities completed or changes in depressive or anxious symptoms. Combining these previous findings with those presented here suggests that 2 aspects of the coached version of Vira, the presence of a human coach combined with the use of nudges toward specific behavior change recommendations, may be responsible for the stronger effects observed in the coached condition in this study. This has implications for future developments of the self-care version of Vira (as well as other groups developing self-guided digital support tools). For example, a feature that allows self-care users to schedule their own in-app nudges or reminders may enhance effectiveness [42] and could also increase scalability by reducing or eliminating the need for a human coach.

Although both intervention conditions likely require fewer resources than traditional face-to-face interventions, the human coaching required some resources, including training and supervision of coaches, time spent in weekly text sessions, and time spent reviewing Vira between sessions and sending personalized behavior nudges or reminders. With the increasing accuracy of transformer-based machine-learning techniques, including via retrieval-augmented generation for large language models [78], developing and testing software features that would support a personalized, fully virtual coached version of Vira is an important next step. Using reinforcement learning with human feedback, a fully virtual coach model could be trained to use a motivational interviewing approach to understand each user’s needs and support personalized goal setting. The virtual coach model could also be trained to recommend specific nudges or reminders based on the user’s passively sensed behavioral data (ie, data on activity, sleep, mobility, and language patterns) and chat conversations with the user. Once developed, the acceptability and effectiveness of a fully virtual coach would need to be compared to those of a human coach. While the support functions of interacting with a human (eg, feeling cared for, respected, and validated) may not be fully replicable by an algorithm, early evidence suggests that fully virtual solutions are acceptable and capable of establishing therapeutic bonds. For example, formative research with a fully automated, cognitive behavioral therapy–based conversational agent showed that users established similar working alliances as patients receiving traditional outpatient cognitive behavioral therapy [79]. Another study comparing experiences with a human coach with those with a virtual chatbot coach found that the chatbot could create a coach-like experience for participants and had the advantage of being persistent and fostering greater participant autonomy by more consistently offering choices and options during the coaching chat session [80].

Moreover, a recent systematic review and meta-analysis of the effectiveness of chatbots on lifestyle behaviors found that chatbot interventions are efficacious for many of the lifestyle behaviors targeted by Vira, such as physical activity and sleep duration [81], although larger studies with more rigorous designs, objective behavioral measures, and longer follow-up are needed. There are a limited number of studies examining whether virtual coach solutions lead to similar improvements in clinical outcomes to those caused by human-supported digital interventions. Finally, further research should seek to understand care providers’ attitudes toward human or virtual coaches and their willingness to incorporate such interventions into care plans. Although this decentralized research study was conducted in a community-based sample of volunteers, we anticipate that most people using Vira will ultimately be referred by a health care provider, regardless of whether on a clinic waiting list, as part of a stepped care model, or in an integrative behavioral health care model. Thus, in these settings, it will be critical to understand providers’ thoughts and concerns about digital and human coaching to ensure broad uptake and reach.

### Limitations

A few methodological limitations must be considered. Due to the pilot nature of this trial, it did not benefit from a control condition, which would have enabled us to rule out placebo, Hawthorne, and regression to the mean effects. Although results revealed that users improved in several self-reported outcomes after 12 weeks of using Vira, the relatively small sample size likely reduced statistical power to detect smaller effect sizes. Further, it is unclear whether positive effects would be sustained beyond 12 weeks, necessitating future studies with longer follow-up periods that could also enable researchers to examine preventive effects.

The small sample size, specificity of the eligibility criteria (ie, risk for obesity and elevated depressive symptoms), and small absolute numbers of men and some racial and ethnic minority groups point to the need for future studies with larger, more representative samples and broader eligibility criteria to validate the present findings and provide data regarding the generalizability of the findings. Notably, there were a limited number of male participants in the sample (n=9 in each group, representing ~25% of the sample). To ensure the intervention was reaching participants who could most benefit from a BA program, participants were required to report clinically significant depressive symptoms (eg, PHQ-8 score≥10). The lower rate of male participants in this sample is consistent with the higher prevalence of major depression among both adult and adolescent female individuals than among male individuals [82], as well as evidence suggesting higher rates of digital mental health app use among female individuals [83]. Nonetheless, the demographics of the current sample highlight both the known difficulties in recruiting and engaging male individuals in mental health research [84] and the importance of developing and implementing recruitment strategies that target underserved populations. In addition, friction in the experience of onboarding with a coach often led to delays between when coached users downloaded Vira and when they started the intervention. This may have impacted retention. Furthermore, as the intervention consisted of multiple components, most of which were not objectively tracked, we could not quantitatively determine which specific features were effective. Identifying the most effective components using more sophisticated experimental designs [85] is an important future direction. Although using an intent-to-treat analytic approach was a strength, 23% (17/73) of follow-up assessment data were imputed, which may have introduced potential bias and affected the reliability of the results. Finally, it should be acknowledged that the lead investigators in this study (albeit not all investigators) are employed by the company that designed and commercially licenses the Vira platform. Independent evaluations will be important in the future, and others should seek to replicate and extend these findings. The Vira platform is generally available for research and clinical apps to facilitate these evaluations.

### Strengths

This study also had several strengths. The fully decentralized trial design supported the recruitment of a heterogeneous community sample, reflecting the complexities and diversity of everyday life and further enhancing the generalizability and external validity of the findings. The sample also included a fairly balanced proportion of Android and iOS users. In addition, the study used validated patient-reported outcome measures, enhancing the reliability and validity of the data collected, as well as the extent to which these findings can be replicated and placed within the broader literature. Finally, despite the importance of this issue for optimizing digital interventions, there are relatively few studies that directly compare coached versus self-guided versions of the same digital intervention within a unified study protocol. For instance, a recent exploratory analysis by Chang and colleagues [86] generated effect sizes for a digital intervention by analyzing and comparing the results of 2 distinct studies of the same tool (unguided vs coached versions); however, inclusion criteria differed between the 2 trials, and there were high rates of missingness in the study of the unguided version of the intervention, presenting potential bias. This study design, which directly compared coached versus self-guided versions, provides insights into who can most benefit from a coaching intervention, supporting a more efficient and targeted allocation of resources to meet individuals’ unique needs. For example, these results suggest that a coached intervention may be more effective than a self-guided intervention for individuals seeking treatment for elevated depression or anxiety symptoms. By contrast, a self-guided intervention may be as effective as or potentially more efficient than a coached intervention for individuals whose primary concern is stress, sleep disturbance or associated impairment, or emotional support. These findings can facilitate stratified care by informing providers’ decisions regarding whether to refer a patient to a self-guided or coached intervention.

### Conclusions

This study found that both versions of the Vira app showed strong engagement compared to benchmarks, and users who received coaching along with the Vira app showed more sustained engagement than those who used the app for self-care. Both versions of Vira were found to be acceptable and showed initial effectiveness in improving various dimensions of behavioral health and quality of life for young adults with depressive symptoms and obesity risk factors; however, users who received coaching reported greater reductions in depressive and anxiety symptoms than self-care users. Both intervention conditions were perceived as useful and easy to use and increased participants’ motivation and confidence for making behavioral changes. Overall, the combination of Vira with coaching was found to be more engaging and effective, but an enhancement to the self-care version of the app may enhance these outcomes for self-guided users. This study represents a unique contribution to the literature, with indicators of feasibility, acceptability, and effectiveness sufficient to support a fully powered trial in a larger sample. To our knowledge, this is the first trial of an app-based intervention using passive mobile sensing to track behavior and deliver personalized insights. Future directions include conducting a fully powered controlled trial with longer follow-up, optimizing the interventions, developing and testing a virtual coach-supported version, and studying salient mediators and moderators of outcome effects using more objective engagement metrics.

## Appendix

Multimedia Appendix 1 CONSORT-EHEALTH checklist (V 1.6.1).

## Abbreviations

BA: behavioral activation
CONSORT: Consolidated Standards of Reporting Trials
GAD-7: Generalized Anxiety Disorder-7
OR: odds ratio
PHQ-8: 8-item Patient Health Questionnaire
PHQ-9: 9-item Patient Health Questionnaire
PROMIS: Patient-Reported Outcomes Measurement Information System
PSS-10: Perceived Stress Scale-10
TAM: Technology Acceptance Model

## Data Availability

The data sets generated during and analyzed during this study are available from the corresponding author on reasonable request.
